# Augmentation of the Riboflavin-Biosynthetic Pathway Enhances Mucosa-Associated Invariant T (MAIT) Cell Activation and Diminishes Mycobacterium tuberculosis Virulence

**DOI:** 10.1128/mbio.03865-21

**Published:** 2022-02-15

**Authors:** Ruchi Jain Dey, Bappaditya Dey, Melanie Harriff, Elizabeth T. Canfield, David M. Lewinsohn, William R. Bishai

**Affiliations:** a Center for Tuberculosis Research, Johns Hopkins University School of Medicine, Baltimore, Maryland, USA; b VA Portland Health Care System, Portland, Oregon, USA; c Division of Pulmonary and Critical Care Medicine, Oregon Health & Science University, Portland, Oregon, USA; University of Pittsburgh

**Keywords:** BCG, MAIT cells, *Mycobacterium tuberculosis*, riboflavin, mucosal immunity, tuberculosis, tuberculosis vaccines

## Abstract

Mucosa-associated invariant T (MAIT) cells play a critical role in antimicrobial defense. Despite increased understanding of their mycobacterial ligands and the clinical association of MAIT cells with tuberculosis (TB), their function in protection against Mycobacterium tuberculosis infection remains unclear. Here, we show that overexpressing key genes of the riboflavin-biosynthetic pathway potentiates MAIT cell activation and results in attenuation of M. tuberculosis virulence *in vivo*. Further, we observed greater control of M. tuberculosis infection in MAIT^hi^ CAST/EiJ mice than in MAIT^lo^ C57BL/6J mice, highlighting the protective role of MAIT cells against TB. We also endogenously adjuvanted Mycobacterium bovis BCG with MR1 ligands via overexpression of the lumazine synthase gene *ribH* and evaluated its protective efficacy in the mouse model of M. tuberculosis infection. Altogether, our findings demonstrate that MAIT cells confer host protection against TB and that overexpression of genes in the riboflavin-biosynthetic pathway attenuates M. tuberculosis virulence. Enhancing MAIT cell-mediated immunity may also offer a novel approach toward improved vaccines against TB.

## INTRODUCTION

Mucosa-associated invariant T (MAIT) cells are a population of innate lymphocyte cells restricted by major histocompatibility complex (MHC) class I-related protein 1 (MR1) (1–3). MAIT cells express an invariant T cell receptor alpha (iTCR-α) chain (iVα7.2-Jα33 in humans and iVα19-Jα33 in mice) and are a critical component of innate host immunity to infections at mucosal sites (1, 4, 5). The discovery that MAIT cells recognize ligands derived from the riboflavin biosynthesis pathway in diverse strains of bacteria and yeast when presented by MR1 led to significant advances in our understanding of the role of MR1 in antigen presentation and MAIT cell activation (6, 7). In fact, activation of MAIT cells exhibits a strong positive correlation with increased production of microbial riboflavin intermediates (8–11).

MAIT cell-mediated antimicrobial immune responses have been reported in a plethora of microbial infections, including Mycobacterium tuberculosis, M. bovis BCG, M. abscessus, Staphylococcus aureus, Salmonella enterica, Legionella longbeachae, Escherichia coli, and Candida albicans (12–26). Ablation of MR1 was found to result in increased susceptibility to a number of bacterial infections (14, 27–29). MAIT cells mediate bacterial control via multiple direct and indirect mechanisms. Cytolytic proteins secreted by MAIT, such as granulysin, kill bacterium-infected cells and also directly kill bacteria by promoting permeabilization of bacterial cells (30, 31). MAIT cells also achieve indirect control of bacteria via recruitment, transactivation, and/or promoting maturation of other antibacterial immune cells (reviewed in reference 31). Recognition of microbial ligands by MAIT cells in mice and humans leads to their activation, resulting in Th1 (primarily gamma interferon [IFN-γ] and tumor necrosis factor alpha [TNF-α]) and Th17 (primarily interleukin 17 [IL-17] and IL-22) proinflammatory cytokine profiles. These mechanisms together exert antimicrobial action against invading pathogens (13, 21, 32–35). MR1-independent MAIT cell activation also occurs in response to stimulation with cytokines, such as IL‐12, IL‐15, IL‐18, and IFN‐α/β (19, 36–40). In a number of acute bacterial infections, patients exhibited a lower number of circulating MAIT cells with features of immune exhaustion, while a higher abundance of activated MAIT cells was found at the site of infection (12, 20, 41–43).

The role of MAIT cells in M. tuberculosis infection has been studied extensively over the last decade, tremendously enriching our understanding of the clinical association of MAIT cells in different forms of tuberculosis (TB) infection or disease, including active pulmonary TB, latent TB, TB-HIV coinfection, childhood TB, and pleural TB (12, 15, 16, 44–49). In addition, modulation of MAIT cell numbers and activation profiles has been observed during the course of treatment of TB and TB-HIV coinfection (12). A majority of studies indicate the presence of a higher number of MAIT cells in the peripheral blood of latently infected individuals (defined by tuberculin skin test positivity [TST^+^] or interferon gamma release assay positivity [IGRA^+^]) and a lower number in patients with active TB disease. Recent epidemiological studies suggest that TST^+^ and IGRA^+^ individuals may actually comprise individuals who have cleared the M. tuberculosis infection yet may continue to show immunoreactivity for more than 9 years beyond the elimination of infection (50), which suggests that MAIT cells may play a critical role in preventing reactivation of latent infection. Alternatively MAIT cells may be recruited from the periphery to the lungs during active TB (51, 52). However, at least one study did not find a correlation of peripheral MAIT cell frequencies with TB disease severity (48). Further, HIV infection also causes a loss of lung-associated MAIT cells which fails to be restored upon initiation of antiretroviral therapy (ART), an observation that may contribute to the increased TB susceptibility in HIV-infected patients (53). The importance of MAIT cells in mycobacterial infections is also highlighted by experiments with MR1-deficient mice that show failure to control growth of M. bovis BCG during the early stages of infection (28, 39). MAIT cells were also found to be activated in response to BCG vaccination in the nonhuman primate model, further suggesting that MAIT cells are subject to modulation by vaccination in a manner similar to conventional T cells (54). However, compared to M. tuberculosis infection, BCG is a poor inducer of MAIT cell responses (54, 55).

Studies in a number of bacterial species have identified the enzymes in the riboflavin-biosynthetic pathway as critical for the production of MAIT cell activating ligands such as 7-hydroxy-6-methyl-8-(1-d-ribityl)lumazine (RL-6-Me-7-OH) and 6,7-dimethyl-8-d-ribityllumazine (RL-6,7-diMe) (6). In addition, 5-amino-6-d-ribitylaminouracil (5-A-RU), an early intermediate in bacterial riboflavin synthesis, has been found to act as a MAIT cell agonist that does not bind to MR1 but forms potent MAIT cell-activating ligands, 5-(2-oxoethylideneamino)-6-d-ribitylaminouracil (5-OE-RU) and 5-(2-oxopropylideneamino)-6-d-ribitylaminouracil (5-OP-RU), via nonenzymatic adduct formation with small molecules, such as glyoxal and methylglyoxal, respectively (7). Accumulation of methylglyoxal and glyoxal in M. tuberculosis-infected macrophages and in human pulmonary TB lesions is a well-known phenomenon (56). Although host cells can neutralize these toxic molecules produced during infection using glyoxalase and reduced glutathione (GSH), M. tuberculosis possesses the ability to quench reduced GSH from the host cells using GSH transporters, thereby depriving the host cells of the ability to neutralize the methylglyoxals produced during oxidative stress during infection (57). Hence, production and accumulation of potent MAIT cell ligands in lung lesions are highly likely to occur during TB disease which may explain the accumulation of MAIT cells in pulmonary lesions of active TB patients. Additionally, intracellular infection by mycobacteria leads to production of a large variety of MR1 ligands such as 7,8-didemethyl-8-hydroxy-5-deazariboflavin (FO), photolumazine I (PLI), and photolumazine III (PLIII), which are formed by reaction of 5-A-RU with molecules derived from other metabolic pathways (24).

Despite an increased understanding of mycobacterial MAIT cell-activating ligands, MAIT cell immunoactivation mechanisms, and the clinical associations of MAIT cells in TB disease, it remains unclear (i) whether increased MAIT cell abundance is protective against M. tuberculosis infection, (ii) whether modulation of riboflavin-biosynthetic pathway genes in M. tuberculosis influences MAIT cell activation and bacterial virulence, and (iii) whether adjuvanting BCG with MR1 ligands may enhance its protective efficacy against M. tuberculosis infection. Riboflavin is the precursor of flavin mononucleotide (FMN) and flavin adenine dinucleotide (FAD), two extremely important cofactors for several hundreds of flavoproteins required for many redox metabolic reactions of physiological importance (58). These cofactors also play role in oxidative stress response (59) and activation of other vitamins such as folate and pyridoxine (60) and hence are important for intracellular survival of pathogenic bacteria in general.

**FIG 1:**
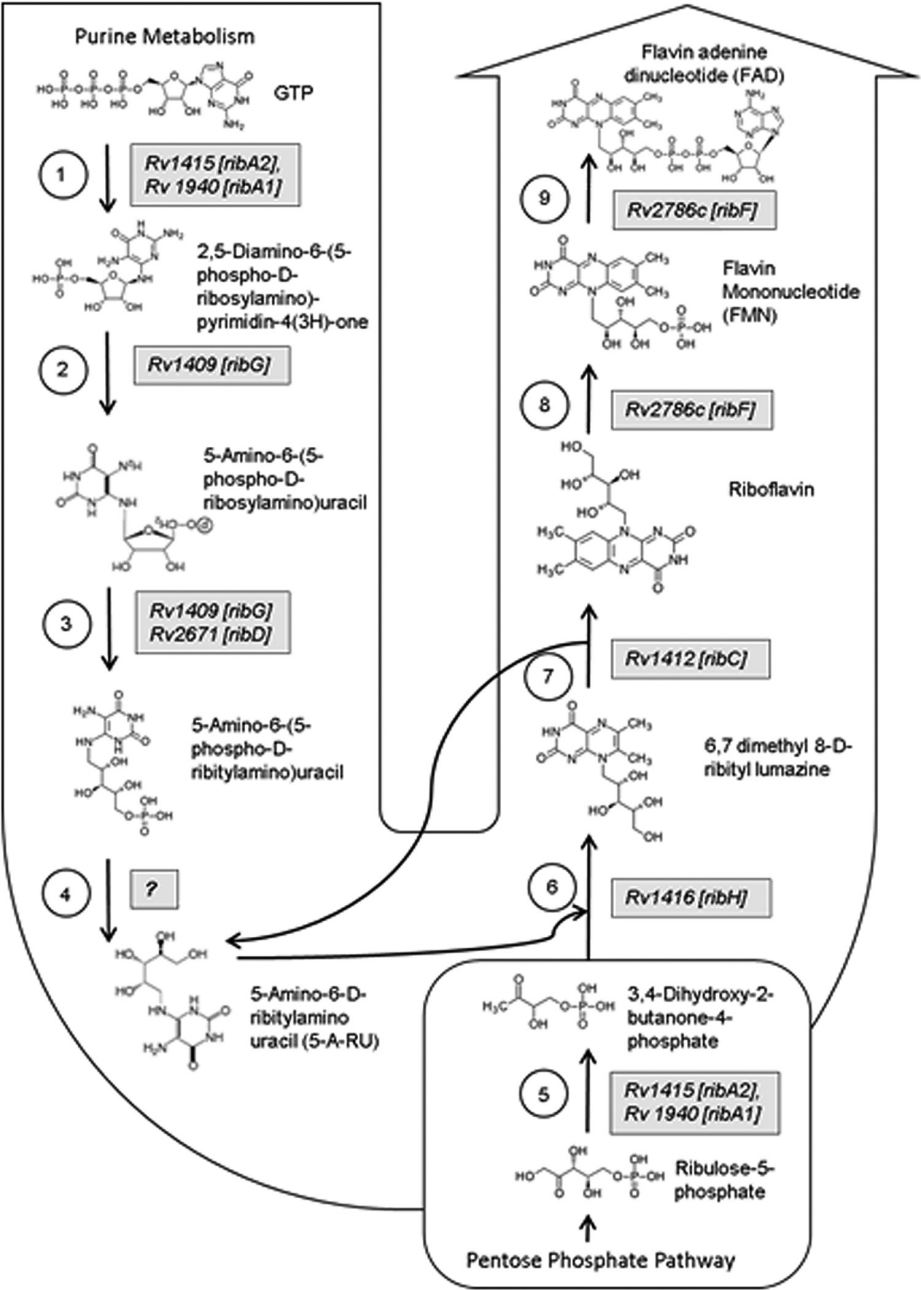
Riboflavin biosynthesis pathway of M. tuberculosis. The flow diagram depicts the enzymatic pathway of riboflavin biosynthesis in M. tuberculosis and the genes [enzymes] involved in various steps. To date, seven genes of M. tuberculosis have been assigned to this pathway. The pathway starts with a bifunctional enzyme, GTP cyclohydrolase II (step 1), which also function as 3,4-dihydroxy-2-butanone 4-phosphate synthase (step 5). Two homologous genes are known to function at this step, *ribA1* and *ribA2.* Steps 2 and 3 are catalyzed by a bifunctional enzyme, RibG, which can function as diaminohydroxyphosphoribosylaminopyrimidine deaminase and 5-amino-6-(5-phosphoribosylamino)uracil reductase. At step 3, two homologs, *ribG* and *ribD*, function to produce 5-amino-6-(5-phosphoribosylamino)uracil, which is the precursor for synthesis of 5-A-RU at step 4. 5-A-RU is a MAIT cell agonist that forms potent ligands on reaction with bacterial and host metabolites such as glyoxals and methylglyoxals. At step 6, another MAIT cell-activating ligand product, 6,7-dimethyl 8-d-ribityl lumazine, is formed by a reaction between 5-A-RU and 3,4-dihydroxy-2-butanone-4-phosphate (obtained from step 5) catalyzed by 6,7-dimethyl-8-ribityllumazine synthase (*ribH*). Lumazine is acted upon by riboflavin synthase (*ribC*) at step 7, producing riboflavin, which is further acted upon by a bifunctional enzyme (*ribF*), which functions at two steps (step 8 and 9), functioning as riboflavin kinase and FMN adenylyltransferase. Of all the genes involved, *ribA2*, *ribG*, *ribH*, and *ribF* have been identified as genes essential for M. tuberculosis survival. The riboflavin-biosynthetic pathway is represented as per the information available in the KEGG pathway (https://www.genome.jp/kegg-bin/show_pathway?mtu00740) and gene essentiality information is based on information available at https://webhost.nts.jhu.edu/target/Default.aspx.

M. tuberculosis possesses an elaborate riboflavin-biosynthetic pathway composed of seven genes: *ribA1*, *ribA2*, *ribC*, *ribD*, *ribF*, *ribG*, and *ribH* (Fig. 1). Key genes in this pathway, *ribA2*, *ribG*, *ribH*, and *ribF*, are essential for *in vitro* and *in vivo* survival of M. tuberculosis as identified by us (https://webhost.nts.jhu.edu/target/Default.aspx) and others (61, 62) by transposon mutant screening (Table S1). These studies further support the metabolic essentiality of riboflavin and cofactors, which possibly cannot be replaced by other cofactors such as iron (63) or be imported by M. tuberculosis from the host, and hence, M. tuberculosis may exclusively depend on endogenous biosynthesis of riboflavin. Proteomic analysis of nonreplicating persistent M. tuberculosis by Cho et al. also showed significant enhancement in expression of ribH during hypoxia (64). However, the roles of the riboflavin-biosynthetic pathway in M. tuberculosis persistence and in counteracting stress encountered inside the host cells are yet to be elucidated. In addition, the mechanisms of genetic control of biosynthesis and transport (including secretion or import) of these metabolites for mycobacterial species are yet unknown.

10.1128/mbio.03865-21.5 TABLE S1 Gene descriptions and primers used in the study. Download Table S1, PDF file, 0.4 MB. Copyright © 2022 Dey et al. 2022 Dey et al. https://creativecommons.org/licenses/by/4.0/ This content is distributed under the terms of the Creative Commons Attribution 4.0 International license.

We hypothesized that upregulating genes in the riboflavin-biosynthetic pathway of M. smegmatis, M. tuberculosis, and M. bovis BCG may potentiate the MAIT cell activation. Hence, in this study, we genetically modified nonvirulent M. smegmatis and virulent M. tuberculosis to constitutively overexpress *ribA2* (*Rv1415*), *ribG* (*Rv1409*), *ribH* (*Rv1416*), and *ribF* (*Rv2786c*) individually. We then assessed the potential of these recombinant mycobacterial strains to activate human MR1-restricted T cell clones by IFN-γ enzyme-linked immunospot (ELISPOT) assay. Subsequently, the *in vivo* growth and virulence of the M. tuberculosis overexpression strain showing the highest MAIT cell activation were evaluated in comparison to wild-type M. tuberculosis in MAIT^lo^ (C57BL6J mice) and MAIT^hi^ (CAST/EiJ mice). We also evaluated the protective efficacy of a recombinant Mycobacterium bovis BCG (BCG Pasteur) strain overexpressing *ribH* (*Rv1416*) to assess if endogenously adjuvanting BCG with MR1 ligands via enhancing riboflavin biosynthesis genes may enhance its protective efficacy.

## RESULTS

### Generation of mycobacterial strains overexpressing key genes in the riboflavin-biosynthetic pathway.

In order to determine the contribution of various riboflavin-biosynthetic enzymes to potentiating MAIT cell activity, a series of genetically modified strains of M. smegmatis and M. tuberculosis were constructed. Of the seven genes mapped so far in the riboflavin-biosynthetic pathway of M. tuberculosis, we constitutively overexpressed four key genes of the riboflavin-biosynthetic pathway essential for *in vitro* and *in vivo* survival of M. tuberculosis, namely, *ribA2* (*Rv1415*), *ribG* (*Rv1409*), *ribH* (*Rv1416*), and *ribF* (*Rv2786c*) individually (Tables S1 to S3). As highlighted in Fig. 1, the RibA2 enzyme functions at key initial steps allowing influx of the primary substrates GTP and ribulose-5-phosphate into riboflavin biosynthesis pathway. Overexpression of *ribA2* would be expected to increase overall upregulation of the pathway by increasing the initial amount of key metabolic intermediates. The RibG enzyme functions after RibA2 at two consecutive steps crucial for production of 5-A-RU; hence, overexpression of *ribG* would be anticipated to overproduce the MAIT cell agonists which form adducts with glyoxals and methylglyoxals, yielding MAIT cell-activating ligands. Alternatively, 5-A-RU may also condense with tyrosine to generate the deazaflavin chromophore FO, which may serve as a MAIT cell-inhibiting ligand depending on the expression levels of FO synthase (24). RibH is the penultimate step of riboflavin synthesis and plays a role in production of 6,7-dimethyl-8-d-ribityllumazine, a known MAIT cell-activating ligand. RibF converts riboflavin into flavin adenine mononucleotide (FMN) and flavin adenine dinucleotide (FAD). Riboflavin accumulation exerts negative feedback on the pathway and is also known to inhibit MAIT cell activation by competing for MR1 binding (24, 65). Hence, *ribF* overexpression would be anticipated to limit the inhibitory effects of riboflavin accumulation, thereby keeping the pathway in an “on” state and ensuring continuous production of intermediary metabolites (66).

We cloned these four *rib* genes under the control of a strong mycobacterial promoter, P*_hsp60_*, in an E. coli-mycobacterial shuttle vector, pSD5hsp60 (67). Recombinant M. smegmatis and M. tuberculosis clones harboring these plasmids were selected and confirmed by PCR and sequencing (Fig. S1 and S2). Notably, M. smegmatis *rib* overexpressing strains displayed a visible yellow pigmentation of the colonies as well as a measurable quantity of riboflavin and lumazine in their culture supernatants (Fig. S3). The M. smegmatis *ribA2* overexpression (OE) strain showed a greater amount of riboflavin and lumazine in the culture supernatant on spectral analysis, suggesting secretion of these metabolites (Fig. S3). Interestingly, while we could obtain M. tuberculosis strains overexpressing the four key genes (*ribA2*, *ribG*, *ribH*, and *ribF*) with no apparent impact on *in vitro* growth, despite our repeated attempts to generate recombinant strains of M. bovis BCG overexpressing these genes, we could only obtain colonies of BCG overexpressing *ribH.* This observation suggests a growth-inhibitory effect of *ribA2*, *ribG*, and *ribF* overexpression in BCG. These mycobacterial *rib*-overexpressing strains were subsequently used for MAIT cell activation studies.

10.1128/mbio.03865-21.1 FIG S1 Characterization of recombinant strains of M. smegmatis. (A) Morphological features of recombinant clones overexpressing *ribA2*, *ribF*, *ribG*, and *ribH* of M. tuberculosis in M. smegmatis. Recombinant bacteria overexpressing (OE) *ribA2* and *ribH* appear to be more pigmented (yellowish) than the wild-type or recombinant strains overexpressing *ribF* or *ribG*. (B) Colonies of each strain were inoculated into 7H9 broth and growth characteristics were determined for recombinant strains with respect to wild-type M. smegmatis. All the strains grew similarly, but RibA2 OE broth cultures were more pigmented (yellowish) than other strains, suggesting greater secretion of the riboflavin or related metabolites of the pathway with characteristic yellow color. (C) Screening of recombinant clones was also performed by colony PCR on DNA isolated from recombinant clones (four each) of *ribA2*-OE, *ribF*-OE, *ribG*-OE, and *ribH*-OE strains using kanamycin resistance gene (*kan*)-specific primers as shown in Table S1. Wild-type M. smegmatis DNA and a no-template control were used as negative controls. DNA from bacteria containing a vector control and a purified vector control plasmid carrying *kan* was used as the positive control. Amplification in clones or the positive control results in a PCR product of 300 bp. Download FIG S1, PDF file, 0.6 MB. Copyright © 2022 Dey et al. 2022 Dey et al. https://creativecommons.org/licenses/by/4.0/ This content is distributed under the terms of the Creative Commons Attribution 4.0 International license.

10.1128/mbio.03865-21.2 FIG S2 Characterization of recombinant strains of M. tuberculosis (a) and M. bovis BCG (b). (a) Screening of recombinant clones of M. tuberculosis overexpressing *ribA2*, *ribF*, *ribG*, and *ribH*. (b) Screening of recombinant clones of BCG overexpressing *ribH*. For screening, colony PCR was performed on DNA isolated from recombinant clones using kanamycin resistance gene (*kan*)-specific primers. DNA isolated from wild-type M. tuberculosis and BCG and a no-template control were used as negative controls. DNA from bacteria containing vector control and/or purified vector control plasmid carrying *kan* was used as the positive control. Amplification in clones or the positive control results in a PCR product of 300 bp. Download FIG S2, PDF file, 0.8 MB. Copyright © 2022 Dey et al. 2022 Dey et al. https://creativecommons.org/licenses/by/4.0/ This content is distributed under the terms of the Creative Commons Attribution 4.0 International license.

10.1128/mbio.03865-21.3 FIG S3 Spectral analysis of culture supernatant of M. smegmatis strains overexpressing genes of riboflavin metabolites between 380 nm and 510 nm. Compared to culture supernatant obtained from wild-type M. smegmatis, culture supernatant obtained from the *ribA2-*OE strain shows higher absorbance between 380 and 475 nm, with an unusually high peak at a wavelength corresponding to the λ_max_ of 6,7-dimethylribityllumazine (∼408 nm) and riboflavin (∼470 nm), suggesting greater production and secretion of these metabolites on overexpression of first gene of the pathway, i.e., *ribA2.* Interestingly, the *ribH-*OE strain, despite showing pigmented colonies (yellowish), did not show an increase in secretion of the metabolites into the broth compared to wild-type M. smegmatis. Download FIG S3, PDF file, 0.4 MB. Copyright © 2022 Dey et al. 2022 Dey et al. https://creativecommons.org/licenses/by/4.0/ This content is distributed under the terms of the Creative Commons Attribution 4.0 International license.

### Enhanced MAIT cell activation by mycobacteria overexpressing key genes of the riboflavin-biosynthetic pathway.

**FIG 2:**
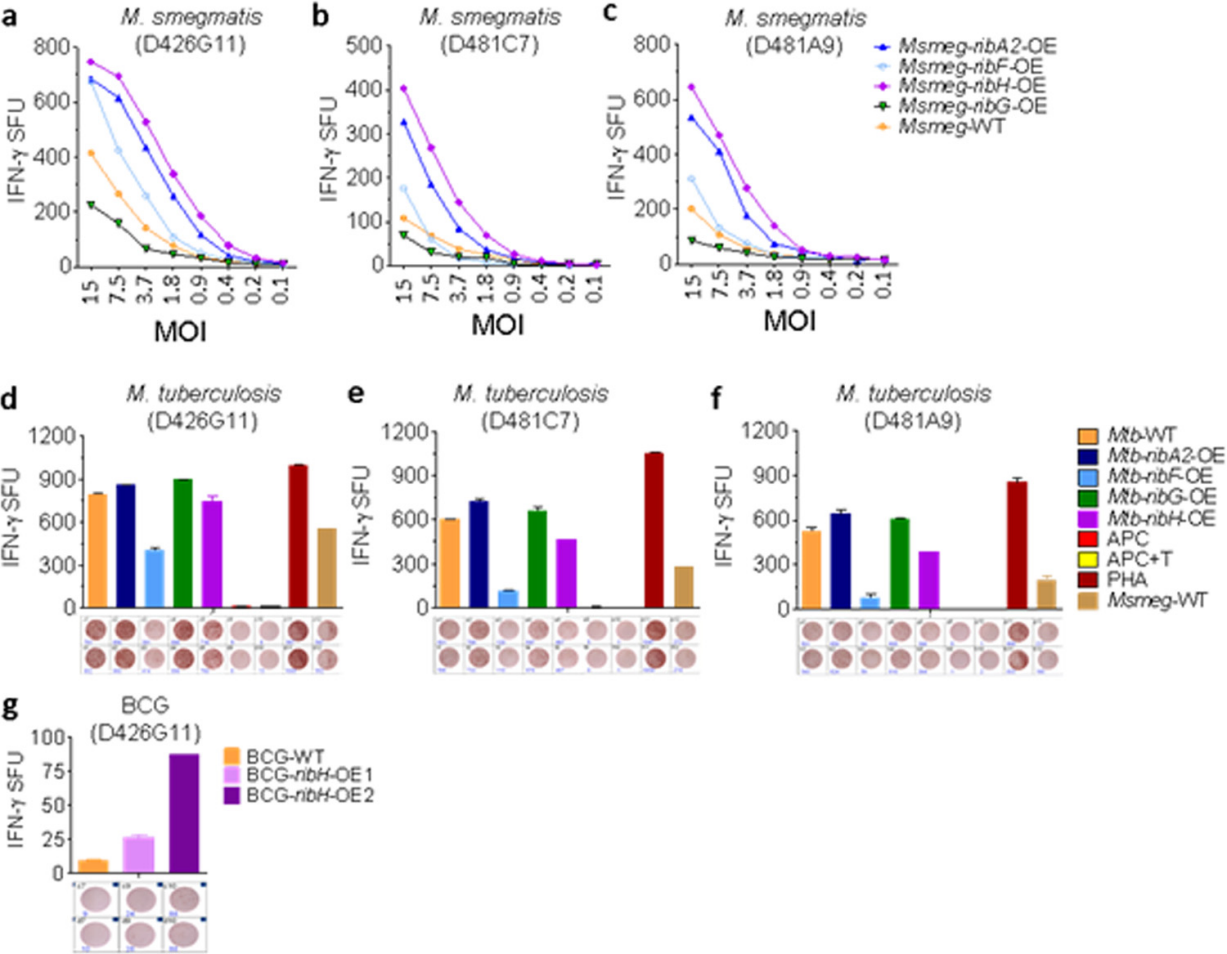
MAIT cell activation by mycobacterial strains overexpressing riboflavin biosynthesis pathway genes. Human bronchial epithelial cells (BEAS-2B) and MR1-restricted CD8^+^ MAIT cells were cocultured in conjunction with mycobacterial infection, and IFN-γ spot-forming units (SFU) were assessed by ELISPOT assay as a readout for MAIT cell activation. The ELISPOT images are presented below the bar diagrams. (a to c) MAIT cell clones D426G11 (a), D481C7 (b), and D481A9 (c) were stimulated by BEAS-2B cells infected with WT and recombinant M. smegmatis overexpressing four genes, namely, *ribA2*, *ribF*, *ribG*, and *ribH* at various MOI from 1:15 (1 BEAS-2B cell to 15 bacilli) to 1:0.1, followed by a serial 2-fold dilution of infecting bacilli. At a calibrated time point, IFN-γ SFU were assessed. Each point in the line diagrams is the mean of duplicate values for each MOI. (d to f) BEAS-2B cells were infected with wild-type and recombinant strains of M. tuberculosis overexpressing four genes, namely, *ribA2*, *ribF*, *ribG* and *ribH*, at the MOI described in Materials and Methods, followed by coculture with three MR1-restricted MAIT cell clones, D426G11 (d), D481C7 (e), and D481A9 (f). IFN-γ SFU data are presented as means and standard deviations (SD) of duplicate values. (g) MAIT cells of clone D426G11 were stimulated by BEAS-2B cells infected with wild-type and recombinant BCG overexpressing *ribH.* Following coculture, IFN-γ SFU were counted. Data are means and SD of duplicate values. APC, antigen-presenting cells; APC + T, antigen-presenting cells plus MAIT cells; PHA, phytohemagglutinin.

To evaluate if overexpression of these key enzymes in the riboflavin biosynthesis pathway has any influence on MAIT cell activation, we performed a coculture assay with the human bronchial epithelial cell line BEAS-2B and MR1-restricted CD8^+^ MAIT cells in the presence of mycobacterial infection. This assay is coupled with IFN-γ ELISPOT formation and serves as a readout of MAIT cell activation, as described previously (24, 43, 54). In these experiments, we included three MR1-restricted MAIT cell clones, namely, D426G11, D481C7, and D481A9, that have been reported to respond to M. tuberculosis-infected cells (24, 43, 54). A series of experiments were carried out using the M. smegmatis-, M. tuberculosis-, and BCG-based overexpression strains. In the first set of experiments, we observed activation of MR1-restricted MAIT cells for wild-type as well as recombinant strains of M. smegmatis that was dependent on the multiplicity of infection (MOI). Notably, all of the OE strains exhibited more IFN-γ spot-forming units (SFU) than wild-type M. smegmatis with the following order: *ribH*-OE > *ribA2*-OE > *ribF*-OE > M. smegmatis (parent strain) > *ribG*-OE. The trend remained similar for all of the MAIT cell clones tested in this experiment (Fig. 2a to c).

Next, we assessed MAIT cell responses to the recombinant M. tuberculosis overexpressing strains. We observed IFN-γ SFU stimulation in the order *ribA2*-OE ≥ *ribG*-OE > WT M. tuberculosis > *ribH*-OE> *ribF*-OE, a pattern somewhat different from that seen with M. smegmatis (Fig. 2d to f). While the magnitude of activation levels differed between the three MAIT T cell clones tested (D426G11, D481C7, and D481A9), the pattern of which overexpressers were strongest (*ribA2*-OE ≥ *ribG*-OE > WT M. tuberculosis > *ribH*-OE> *ribF*-OE) remained consistent.

In the third set of experiments, we measured MAIT cell responses following BCG stimulation. We tested WT BCG and two clones of the *ribH*-OE BCG strain. While both clones considerably augmented the MAIT cell IFN-γ ELISPOT response compared to WT BCG, BCG overexpressing *ribH* clone 2 enhanced the response by 9.3-fold (Fig. 2g).

### Overexpression of riboflavin metabolites reduces M. tuberculosis virulence *in vivo* in the mouse TB model.

**FIG 3:**
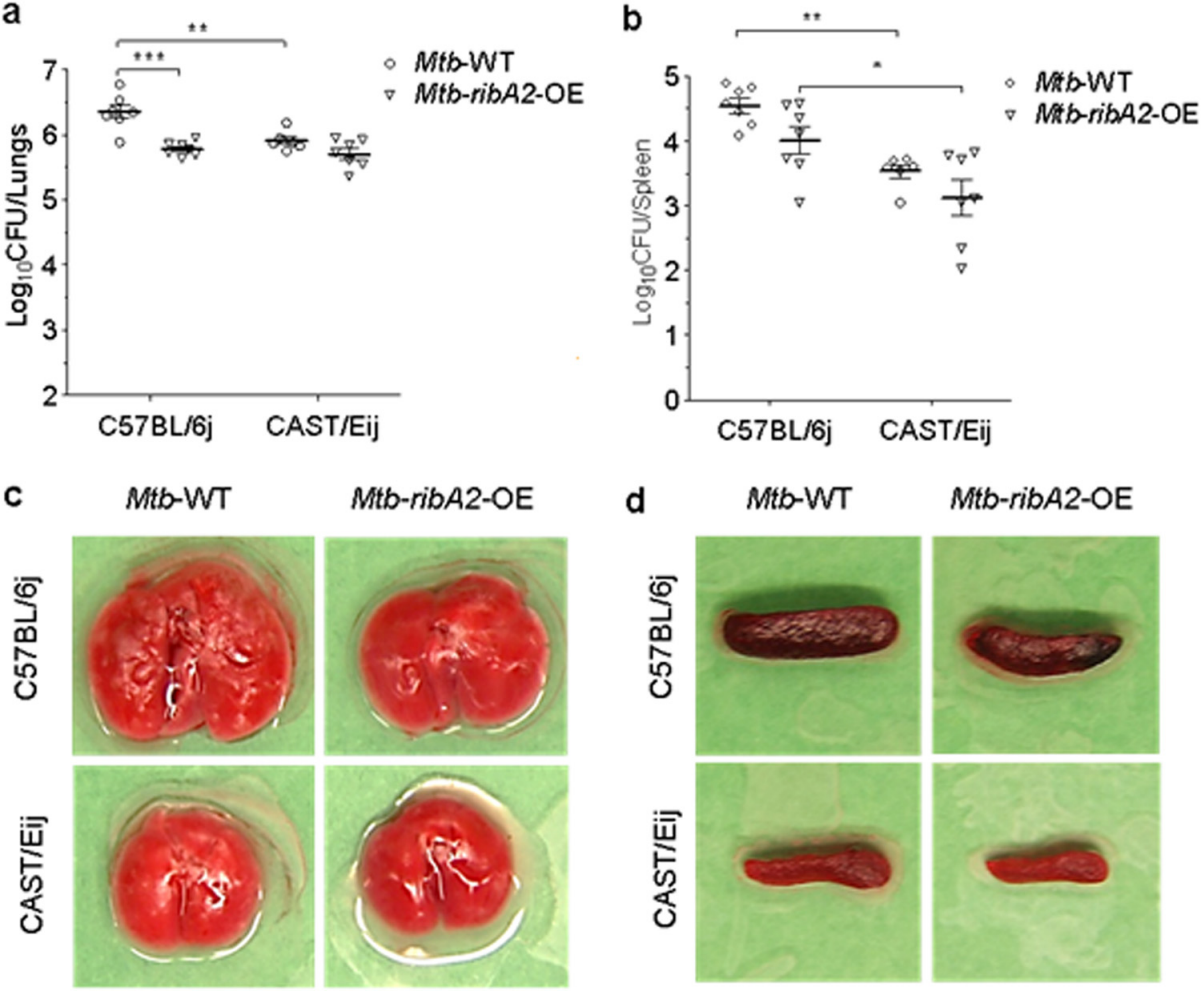
Overexpression of *ribA2* attenuates M. tuberculosis in a mouse model, and MAIT^hi^ mice are inherently more resistant to M. tuberculosis infection. Bacterial growth and gross pathological features of lungs and spleens were evaluated 5 weeks following aerosol infection with WT and *ribA2*-OE M. tuberculosis strains in C57BL/6J (MAIT^lo^) and CAST/EiJ (MAIT^hi^) mice. (a and b) Bacillary load in lungs (a) and spleens (b), presented as log_10_ CFU. Data are means and standard errors of the means (SEM) (*n *= 7). *, *P < *0.05; **, *P < *0.01; ***, *P < *0.001 (one-way ANOVA with Tukey’s posttest). (c and d) Gross pathological features of lungs (c) and spleens (d) of representative mice from each group are depicted.

We then tested whether enhancing the levels of riboflavin metabolites during M. tuberculosis infection contributes to greater control of TB disease. We compared the virulence of the M. tuberculosis *ribA2*-OE strain (which showed the highest MAIT cell activation *in vitro* [Fig. 2d to f]) to that of WT M. tuberculosis in MAIT^lo^ (C57BL6J) mice, as per the study design described in Fig. S4. RibA2 overexpression resulted in a marked reduction of M. tuberculosis proliferation, as evidenced by reduced lung and spleen bacterial loads compared to WT M. tuberculosis at 5 weeks postchallenge (Fig. 3a and b). Notably, the extent of reduction in lung and spleen CFU of the *ribA2*-OE strain versus the WT was 3.7-fold and 3.4-fold, respectively. We also observed a reduction in the gross pathology of lungs and spleens, with reduced tubercle numbers and sizes in the lungs of mice infected with the *ribA2*-OE strain compared to WT-infected mice (Fig. 3c and d). Together, these observations clearly indicate that MAIT^hi^ mice are inherently more resistant to M. tuberculosis infection and that *ribA2* overexpression leads to attenuation of virulence of M. tuberculosis.

10.1128/mbio.03865-21.4 FIG S4 (a) Design of the virulence study wherein virulence of WT and *ribA2*-OE M. tuberculosis was compared in MAIT^lo^ (C57BL/6J) mice and in MAIT^hi^ (CAST/EiJ) mice at 5 weeks postinfection. (b) Design of vaccination studies carried out in C57BL/6J mice, wherein vaccine efficacy of *ribH*-OE BCG was tested against aerosol infection with WT M. tuberculosis. Download FIG S4, PDF file, 0.5 MB. Copyright © 2022 Dey et al. 2022 Dey et al. https://creativecommons.org/licenses/by/4.0/ This content is distributed under the terms of the Creative Commons Attribution 4.0 International license.

### Mice with intrinsically higher MAIT cell abundance show improved M. tuberculosis containment in the mouse TB model.

MAIT cells are abundant in humans (5 to 10% of total circulating T cells); however, MAIT cells are rare in common laboratory mouse strains such as C57BL/6J and BALB/c (∼0.1% of T cells in lymphoid organs) (35, 68, 69). So far, the study of MAIT cell physiology has mostly relied on mice that are transgenic for the MAIT cell TCR chains, allowing the study of MAIT cells *in vitro* and *in vivo* (5, 70, 71). In non-TCR-transgenic mouse models, antibody-mediated or genetic depletion of conventional T cells has been used to elucidate the role of MAIT cells *in vivo* (18, 29). A notable study by Cui et al. showed that specific-pathogen-free wild-derived inbred CAST/EiJ mice possess a significantly higher frequency of MAIT cells (∼20 times) than C57BL/6J mice and, thus, may represent a useful small-animal model to evaluate the role of these naturally occurring mouse MAIT cells in health and disease and to evaluate vaccines and therapies targeting MAIT cells (72).

Thus, we next assessed whether elevated MAIT cell abundance improves host containment of M. tuberculosis proliferation in the mouse TB model. To address this, we performed a comparative assessment of M. tuberculosis growth in CAST/EiJ (MAIT^hi^) mice, which naturally express significantly higher frequencies of MAIT cells (∼20 times) than C57BL/6J (MAIT^lo^) mice (Fig. S4). As may be seen in Fig. 3a and b, compared to C57BL/6J (MAIT^lo^) mice, CAST/EiJ (MAIT^hi^) mice exhibited better control of lung and spleen bacterial proliferation, with 0.44 log_10_ unit fewer CFU (2.7-fold) in the lungs and 1.0 log_10_ unit fewer CFU (10.3-fold) in the spleen 5 weeks following aerosol challenge (Fig. 3a and b). Gross pathology analysis of lung and spleens recapitulated the bacteriological observations, with markedly fewer and smaller tubercles in the lungs and no evident sign of splenomegaly in CAST/EiJ (MAIT^hi^) mice compared to C57BL/6J (MAIT^lo^) mice (Fig. 3c and d). In fact, the spleens in the former mice were relatively much smaller and less inflamed than in the latter strain.

We also tested if MAIT^hi^ mice offer any further improvement in control of infection in case of the M. tuberculosis *ribA2*-OE strain. However, the extent of reduction in lung and spleen CFU of the *ribA2*-OE strain versus WT was greater in MAIT^lo^ mice than in MAIT^hi^ mice (lung CFU, 3.7-fold versus 1.6-fold; spleen CFU, 3.4-fold versus 2.5-fold) (Fig. 3a and b). In terms of pathology, reduced tubercle numbers and sizes in lungs and no evident sign of splenomegaly were observed in mice infected with the *ribA2*-OE strain, and the extent of reduction in pathology was greater in MAIT^hi^ mice than MAIT^lo^ mice (Fig. 3c and d). Together, these observations clearly show that MAIT^hi^ mice are inherently more resistant to M. tuberculosis infection and that *ribA2* overexpression leads to attenuation of virulence of M. tuberculosis.

### Protective efficacy of recombinant BCG overexpressing *ribH*.

**FIG 4:**
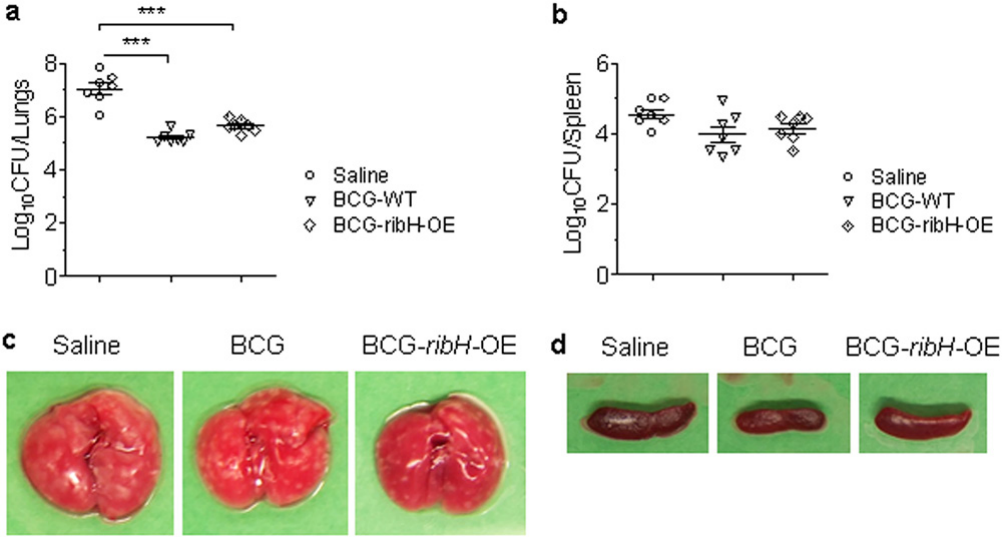
Vaccine efficacy of BCG overexpressing *ribH* against M. tuberculosis infection in mice. (a and b) Bacillary load in the lungs and spleens of mice vaccinated with saline control, BCG, and *ribH*-OE BCG by the subcutaneous route. At 6 weeks following vaccination, mice were infected with M. tuberculosis by the aerosol route. At 5 weeks postinfection, mice (*n* = 7) were euthanized, and lung (a) and spleen (b) bacillary loads and gross pathology were assessed. Data are means and SEM (*n *= 7). ***, *P < *0.001 by one-way ANOVA with Tukey’s posttest. (c and d) Gross pathological features of lungs (c) and spleens (d) of a representative mouse from each group.

As BCG is a poor inducer of MAIT cell responses, we next evaluated if endogenously enriching BCG with MAIT ligands via overexpression of riboflavin biosynthesis genes may enhance its protective efficacy against M. tuberculosis infection. Despite our repeated attempts to generate recombinant strains of M. bovis BCG overexpressing the *ribA2*, *ribG*, and *ribF* genes, we could only obtain colonies of BCG overexpressing *ribH.* This observation most likely suggests a lethal effect of overexpressing *ribA2*, *ribG*, and *ribF* on BCG survival *in vitro*. Since overexpression of *ribH* in BCG moderately enhanced MAIT cell activation (Fig. 2g), we tested its vaccine efficacy in C57BL/6J mice against M. tuberculosis infection. Six weeks after immunization with BCG and *ribH*-OE BCG, mice were challenged with M. tuberculosis by the aerosol route, and 5 weeks later, lung and spleen bacillary loads and gross pathology were evaluated (Fig. S4). Compared to sham immunization (controls), vaccination with WT BCG and *ribH-*OE BCG resulted in significantly lower bacillary loads in the lungs (1.8 log_10_ units and 1.4 log_10_ units, respectively) and in the spleens (0.57 log_10_ units and 0.4 log_10_ units, respectively) (Fig. 4b and c). The reduction in bacillary loads was also accompanied by reduced gross pathology in lungs and spleens with both BCG and *ribH*-OE BCG compared to the sham-immunized group. However, compared with WT BCG, the *ribH*-OE BCG strain did not significantly improve TB disease containment in this mouse model.

## DISCUSSION

Human MAIT cells restricted by the evolutionarily conserved MHC-like molecule MR1 are known for their ability to detect a wide variety of bacterial and fungal organisms, including M. tuberculosis (15, 18). Increased susceptibility to several bacterial infections, including M. bovis BCG and M. abscessus, in MR1 deletion mutant mice also highlights the important role of MAIT cells in antimicrobial immunity (18). The absence of MAIT cells in germfree mice and the expansion of MAIT cells in response to microbes with an intact riboflavin biosynthesis pathway suggested a close link between bacterial riboflavin-biosynthetic machinery and MAIT cell responses (3, 73–75). These initial observations were followed by a series of studies leading to the discovery of MR1 binding ligands which originate from the riboflavin-biosynthetic pathway. Later, neo-antigens were also discovered; these are biochemical adducts formed between intermediates of the riboflavin pathway and glyoxals or methylglyoxals produced by microbes and the infected host cells experiencing oxidative stress.

Neo-antigens are potent MR1 binders and MAIT cell activators (6, 7, 76). Notably, a recent study identified a broad array of novel MR1 ligands produced during mycobacterial infection, including some which do not appear to originate from the riboflavin biosynthesis pathway (24). For example, intracellular infection by mycobacteria leads to production of MR1 ligands such as 7,8-didemethyl-8-hydroxy-5-deazariboflavin (FO), photolumazine I (PLI), and photolumazine III (PLIII), which are formed by reaction of 5-A-RU with molecules derived from other metabolic pathways (24). FO is a precursor to coenzyme F_420_ and is generated by reaction of 4-hydroxyphenylpyruvate (from the tyrosine biosynthesis pathway) with 5-A-RU, catalyzed by the FO synthase enzyme. Further, PLI and PLIII, which share a core ribityllumazine structure with RL-6,7-diMe, are predicted to be products of reactions between 5-A-RU and α-ketoglutarate (24). These ligands can be distinguished by different T cell receptors on MR1-restricted MAIT cells and possess either activating or inhibitory properties (24). It is speculated that certain unique ligand signatures within the mycobacterial MR1 ligandome may in fact serve as specific pathogen-associated molecular patterns (PAMPs) that allow the immune system to distinguish mycobacterial infections from other microbial infections.

MAIT cells display several features which indicate their possible role during M. tuberculosis infection: (i) MAIT cells numbers and activation profiles are different in humans with latent infection versus active TB disease (49); (ii) MAIT cells can rapidly detect cells infected with M. smegmatis (24), M. bovis BCG (14, 39), M. abscessus (18), and M. tuberculosis (43), leading to production of a large variety of proinflammatory cytokines, such as IFN-γ, TNF-α, and IL-17, and ultimately lysis of the infected cells (13); (iii) MAIT cells display tissue homing and residency in the human respiratory tract, the primary site of M. tuberculosis infection (44, 77); and (iv) single nucleotide polymorphisms (SNPs) in the MR1 gene have been shown to be associated with the risk of TB meningitis (45). A recent study in macaques showed that MAIT cells are subject to transient modulation by vaccination with BCG (78). Another study, in cynomolgus monkeys, showed that prior infection with simian immunodeficiency virus (SIV) results in increased susceptibility to M. tuberculosis infection due to immune exhaustion of MAIT cells (42). Thus, MAIT cells serve as an early source of the cytokines essential for shaping downstream adaptive immunity to infections (79). Moreover, analogous to conventional T cells, MAIT cells have also been found to contribute to the development of immunological memory (43, 80). Since MAIT cells are well suited for controlling infection at the pulmonary mucosal surface, fine-tuning of MAIT cell behavior using a suitable vehicle to deliver MR1 ligands to this compartment may constitute a valuable strategy for developing MAIT cell-based vaccines (55, 81, 82).

In this study, we dissected the riboflavin biosynthesis pathway of M. tuberculosis and related mycobacteria to address some of the aforementioned unanswered questions. Our study used systematic genetic engineering of virulent M. tuberculosis, avirulent M. smegmatis, and the vaccine strain M. bovis BCG to overexpress key genes of the riboflavin pathway. Our re-engineered mycobacteria produced riboflavin metabolites in greater amounts and delivered these antigens intracellularly during infection. *In vitro* assays performed on bronchial epithelial cells infected with these recombinant strains clearly showed that intracellular infection with these metabolically loaded bacteria is successful in delivering MAIT ligands to MR1 with downstream activation of MR1-restricted MAIT cells. In contrast to M. smegmatis, M. tuberculosis does not secrete MR1 ligands, as has been shown by a lack of activation of MR1-restricted MAIT cells upon stimulation with M. tuberculosis culture supernatant (24). This observation indicates that during M. tuberculosis infection, microbial ligands are sampled and loaded intracellularly onto MR1 (83). Hence, endogenous enrichment of mycobacterial MAIT cell ligands may offer a potential strategy to deliver small molecules to create potential vaccines which target MAIT cell activation. In support of this hypothesis, a previous study from our group showed that overexpression of the small-molecule STING agonist cyclic di-AMP could be delivered intracellularly using BCG as a vehicle, leading to substantially augmented BCG-induced anti-TB immunity in the guinea pig model (84). Here, we overexpressed *ribH* in BCG, anticipating enhanced MR1 loading and a resultant increase in MAIT cell activation. While BCG overexpressing *ribH* showed enhanced MAIT cell activation compared to wild-type BCG, it did not achieve the level of activation observed with M. tuberculosis (Fig. 2d to f versus Fig. 2g). This may possibly stem from the fact that compared to M. tuberculosis, methylglyoxals produced by macrophages during BCG infection are lower in abundance (57, 85), which may reduce the level of MAIT cell ligand substrates and result in poorer MAIT cell induction by BCG or its recombinant strains.

Our findings that CAST/EiJ mice, which naturally produce ∼20-fold-higher numbers of MAIT cells than conventional C57BL/6J mice, showed improved control of M. tuberculosis proliferation and granulomatous pathology clearly signifies a possible association of MAIT cells with protection against TB disease. In addition, the attenuation of M. tuberculosis overexpressing *ribA2* observed in this study further supports the hypothesis that bacteria can be metabolically engineered to deliver MAIT cell ligands and that this may arm the host immunity to afford greater control of M. tuberculosis and its pathology, possibly via MAIT cell repertoire expansion or activation. As MAIT^hi^ mice do not offer any further control in TB infection in the case of a RibA2 overexpression strain, future studies measuring the changes in MAIT cell numbers and its activation status *in vivo* in MAIT^hi^ versus MAIT^lo^ mice with these strains could potentially determine if the MAIT^hi^ mice exhibit any further enhancement in MAIT cell number and activity on RibA2 overexpression or if there is a “ceiling” effect in terms of MAIT cell number or activation status with respect to their efficacy against infection. Recent studies highlight the possibility of immune exhaustion and impairment of MAIT cell functions on chronic activation and engagement that may cause these cells to shift from protective to nonprotective functions, for example, in the case of HIV (41), hepatocellular carcinoma (86), and 5-OP-RU-based vaccination (55). Alternatively, there may exist a yet-unidentified MAIT cell-independent protective mechanism in these mice.

Despite a growing knowledge base regarding MR1 ligands, the pathway forward to optimally exploit MAIT cells in the effort to develop improved vaccine formulations remains uncertain. A recent study showed that the potent MR1 ligand 5-OP-RU induces efficient MAIT cell responses only when delivered in conjunction with TLR ligands as costimulating agents (36). This study also suggested induction of a transcription profile characteristic of activation of repair mechanisms in the infected tissues. However, the unstable nature of the MR1 ligands has thus far hindered the development and testing of numerous MR1 ligands for their immunoadjuvant, vaccine, or therapeutic potential (55, 82, 87). These ligands could profoundly benefit vaccine development against infections if combined with potent immunodominant epitopes against specific bacterial infections (81). Such vaccine formulations would allow augmentation of mucosal immunity against specific infections via acquisition of both acquired immunity against specific epitopes and innate immunity from MAIT cells.

Because BCG is a weaker MAIT cell activator than M. tuberculosis (as shown in this study) and other bacteria such as Francisella tularensis LVS (88), and because BCG vaccination activates MAIT cells mainly via an MR1-TCR signaling-independent mechanism (39), we hypothesized that a recombinant BCG which overexpresses MAIT cell ligands might show enhanced protection against TB via improved MR1-TCR signaling-dependent MAIT cell activation and MR1-TCR-independent innate and adaptive immunity generated in response to antigens of BCG. Although our *ribH*-OE BCG strain enhanced MAIT cell activation *in vitro* in coculture assays, the strain did not demonstrate protection beyond that conferred by the parental WT BCG strain. One potential explanation for this observation is that *ribH* overexpression may have promoted more rapid clearance of the recombinant *ribH*-OE BCG following immunization, leading to only modest protection. The probable lethality on overexpressing of riboflavin pathway genes in BCG and no further improvement in vaccine efficacy might also stem from the lack of an ESAT-6 secretion system (ESX-1) encoded by region of difference 1 (RD-1) in BCG (89–92). RD-1 mutation has been shown to contribute to virulence attenuation and reduced tissue invasiveness and cytolysis of pneumocytes and macrophages (92, 93). The ESX-1 secretion system is utilized by M. tuberculosis to secrete a wide variety of virulence factors and metabolites to evade host immunity (94). It is possible that due to the lack of a riboflavin secretion system, metabolites of this pathway accumulate on overexpression of riboflavin genes with consequent lethality. Additionally, reduced or absent export of these metabolites by BCG inside the macrophages might also underlie the observed weaker MAIT cell activation. Future studies on riboflavin secretion system and application of M. tuberculosis auxotrophic mutants with intact ESX-1 system might offer novel ways to develop vaccines targeting MAIT cells.

In summary, the findings of this study demonstrate that MAIT cells play a key role in protection against M. tuberculosis *in vivo*. We also demonstrate that overexpression of the *ribA2* gene in M. tuberculosis leads to greater MAIT cell activation *in vitro* and concomitantly to improved host containment of M. tuberculosis during murine infection *in vivo*. While our *ribH*-OE BCG strain was not superior to WT BCG as a TB vaccine in the mouse model, our data nevertheless support the concept that recombinant mycobacteria overexpressing riboflavin biosynthesis genes elicit more potent MAIT cell responses. Future studies examining the levels of MAIT ligands and the *in vivo* responses they elicit may offer insights valuable for the development of newer tools that exploit MAIT cell-mediated immune control of TB. In addition, alternative methods need to be explored to overexpress riboflavin pathway genes in attenuated mutants of M. tuberculosis, BCG, or probiotic bacteria that may further enhance MAIT activation and confer greater protection against M. tuberculosis infection.

## MATERIALS AND METHODS

### Bacterial strains and culture conditions.

In this study, we used Mycobacterium tuberculosis CDC1551, M. smegmatis mc^2^155 (95–97), and Mycobacterium bovis bacillus Calmette-Guérin (BCG) Pasteur (a generous gift from Frank Collins [FDA] and identical to BCG Pasteur provided by the Pasteur Institute to the Trudeau Institute in 1967 as TMC no. 1011). M. smegmatis strain mc^2^155 has been referred to as the wild type for the purpose of simplifying nomenclature of the recombinant strains developed therefrom in the study from this parent strain. The strain was originally reported by Panas et al. and was shown to carry a mutation in *eptC*, the loss of which conferred the property of efficient plasmid transformation to M. smegmatis and hence provided a fast-growing surrogate model organism as a parent strain to study fundamental cellular processes in mycobacteria (97). Various mycobacterial strains were cultured as described (84); briefly, glycerol stocks were plated on 7H11 plates supplemented with oleic acid-albumin-dextrose-catalase (OADC) (catalog no. B11886; Fisher Scientific). Single isolated bacterial colonies were picked and propagated in 7H9 Middlebrook liquid medium (catalog no. B271310; Fisher Scientific) supplemented with OADC (catalog no. B11886; Fisher Scientific), 0.5% glycerol (catalog no. G55116, Sigma), and 0.05% Tween 80 (catalog no. BP338; Fisher Scientific).

### Generation of recombinant mycobacterial strains.

For cloning of mycobacterial riboflavin pathway genes, genomic DNA from M. tuberculosis strain CDC1551 was used for PCR amplification of riboflavin-biosynthetic pathway genes, namely, *ribA2* (*Rv1415*, *MT1458*), *ribG* (*Rv1409*, *MT1453*), *ribH* (*Rv1416*, *MT1459*) and *ribF* (*Rv2786c*, *MT2856*) using primers listed in Table S1. Cloning experiments were performed using E. coli strain DH5-α (catalog no. 18258012; Fisher Scientific), which was routinely maintained in LB broth. For generation of genetically engineered mycobacterial strains overexpressing riboflavin pathway genes, an E. coli-mycobacterial shuttle vector (pSD5hsp60) was used to clone *ribA2*, *ribF*, *ribG*, and *ribH* under the control of the strong mycobacterial hsp60 promoter as described earlier (98). Clones were confirmed by restriction digestion and gene sequencing and were used for transformation by electroporation into M. smegmatis mc^2^155, M. tuberculosis CDC1551, or M. bovis BCG (Pasteur strain) electrocompetent cells. Recombinant strains were selected on kanamycin (25 μg/mL) and confirmed using colony PCR against the kanamycin resistance cassette (Fig. S1 and S2; Table S1). Details of all the plasmids constructed in the study and recombinant bacterial strains are listed in Tables S2 and S3, respectively.

10.1128/mbio.03865-21.6 TABLE S2 Plasmids used in the study. Download Table S2, PDF file, 0.5 MB. Copyright © 2022 Dey et al. 2022 Dey et al. https://creativecommons.org/licenses/by/4.0/ This content is distributed under the terms of the Creative Commons Attribution 4.0 International license.

10.1128/mbio.03865-21.7 TABLE S3 Bacterial strains used in the study. Download Table S3, PDF file, 0.7 MB. Copyright © 2022 Dey et al. 2022 Dey et al. https://creativecommons.org/licenses/by/4.0/ This content is distributed under the terms of the Creative Commons Attribution 4.0 International license.

### Spectral analysis of culture supernatant of M. smegmatis strains for metabolite analysis.

Due to evident differences in the colony pigmentation and broth cultures of M. smegmatis recombinant strains upon overexpression of riboflavin-biosynthetic pathway genes (Fig. S1a and b), culture supernatants obtained from M. smegmatis strains were evaluated for increased production of riboflavin and 6,7-dimethylribityllumazine by measuring absorbance between 380 nm and 510 nm. An unusually high peak was observed at a wavelength corresponding to 408 nm and 470 nm (corresponding to the λ_max_ of 6,7-dimethylribityllumazine [∼408 nm] and riboflavin [∼470 nm], respectively) (Fig. S3). However, no change in colony morphology was observed in M. tuberculosis or M. bovis BCG recombinant strains.

### BEAS-2B and MAIT cell coculture infection assay.

In this study, we employed three MR1-restricted nonclassical human CD8^+^ MAIT cell clones, namely, D426G11, D481C7, and D481A9, that have been reported to respond to M. tuberculosis-infected cells. As described by Gold et al. (43), these MAIT cell clones were derived from (previously) M. tuberculosis*-*infected donors. D426 was healthy but had latent infection; D481 had an active infection but was treated and was smear negative before the peripheral blood mononuclear cells (PBMC) were collected (43, 99). All the protocols were approved by the Institutional Review Board at Oregon Health and Science University. The three clones have unique TCR rearrangements. These T-cell clones were expanded in the presence of irradiated allogeneic PBMC (25 × 10^6^), a lymphoblastoid cell line (LCL; 5 × 10^6^), and anti-CD3 monoclonal antibodies (MAb) (30 ng/mL) in RPMI 1640 medium with 10% human serum in a T-25 upright flask in a total volume of 30 mL. The cultures were supplemented with IL-2 (0.5 ng/mL) on days 1, 4, 7, and 10 of culture. The cell cultures were washed on day 4 to remove remaining soluble anti-CD3 MAb as described previously (43) and used no earlier than day 11.

A human bronchial epithelial cell line (BEAS-2B) purchased from ATCC (CRL-9609) were grown as described previously (99). These cells are MHC-II-negative, large alveolar epithelial cells, can be infected with M. tuberculosis, and have been shown to stimulate M. tuberculosis-specific CD8^+^ T cells. These BEAS-2B cells are used as an antigen-presenting cells in an IFN-γ ELISPOT assay. In all the BEAS-2B–MAIT cell coculture assays, 10^4^ BEAS-2B cells were cultured with 10^4^ T cells per well.

For assays using wild-type or engineered M. smegmatis, M. tuberculosis, or BCG, all the mycobacterial strains were grown as described above and live infection of BEAS-2B cells was carried out using various multiplicities of infection (MOI) followed by coculture with MAIT cells. In the case of M. smegmatis, after seeding antigen-presenting cells (APC) on ELISPOT plates, bacteria are added like an antigen, and infection is allowed for 1 h, followed by addition of MAIT cells and overnight incubation. The MOI used for M. smegmatis is 1 epithelial cell to 0.1 to 15 bacteria.

For M. tuberculosis or BCG, BEAS-2B cells were plated in 6-well tissue culture plates and infected overnight at an MOI of 1 epithelial cell to 5 bacteria. After 16 h, infected cells were harvested using RPMI (Gibco) supplemented with l-glutamine, 10% heat-inactivated human serum, and gentamicin. The cells were used as antigen-presenting cells in an IFN-γ ELISPOT assay (10,000 cells/well, plated in duplicate). ELISPOT assays were done using 96-well mannose-sensitive hemagglutinin (MSHA) plates (Merck Millipore) coated with a monoclonal antibody against IFN-γ (Mabtech). After 1 h, the MR1-restricted T cell clones were added at 10^4^ T cells per well. After overnight incubation at 37°C and 5% CO_2_, the plate was developed with alkaline phosphatase antibody (Mabtech). IFN-γ spot-forming units (SFU) were measured on an AID ELISPOT reader (Autoimmun Diagnostika GmbH).

### Animals.

Experimental procedures involving live animals were carried out in agreement with the protocols approved by the Institutional Animal Care and Use Committee (IACUC) at The Johns Hopkins University School of Medicine. For animal infection protocols, pathogen-free 4- to 6-week-old female C57BL/6J and CAST/EiJ mice (The Jackson Laboratory) were purchased and housed under pathogen-free conditions at an animal biosafety level 3 animal facility in individually ventilated cages. Animals were given free access to water and standard chow and were monitored daily for general behavior and appearance by veterinary specialists.

### Animal experiments. (i) Virulence studies in MAIT^hi^ and MAIT^lo^ mouse strains.

We compared the *in vivo* virulence of the M. tuberculosis *ribA2*-OE strain that showed the highest MAIT cell activation in our *in vitro* assays in MAIT^lo^ mice to test if enhancing the levels of riboflavin metabolites during M. tuberculosis infection contributes to greater control of TB disease. For this, age-matched female mice of both MAIT^lo^ strains (10 per group) were infected with the same dose of *ribA2*-OE M. tuberculosis strains by the aerosol route in a Glas-Col inhalation chamber.

We also assessed if the CAST/EiJ mice (MAIT^hi^), which naturally possess a significantly higher frequency of MAIT cells (∼20 times) than C57BL/6J mice (MAIT^lo^), have a greater ability to control M. tuberculosis. For this, age-matched female mice of both strains (10 per group) were infected with equivalent doses of M. tuberculosis or M. tuberculosis *ribA2*-OE strains (∼200 to 400 bacilli) by the aerosol route in a Glas-Col inhalation chamber. Lungs from one set of infected animals (*n* = 3) were harvested from each group, and homogenates were plated on day 1 after infection to check for established implantation. Infected animals from each group (*n* = 7) were euthanized at 5 weeks postinfection to determine the bacillary load. Gross pathological features of lungs and spleens were also compared as described previously (84). Lungs and spleens were harvested and homogenized, and bacillary load was determined by plating serial dilutions of homogenate on 7H11 agar plates supplemented with OADC (catalog no. B11886; Fisher Scientific) as described previously (84).

### (ii) Vaccine efficacy study of *ribH*-OE BCG.

To test the vaccine efficacy of *ribH*-OE BCG, C57BL/6J mice (10 per group) were immunized subcutaneously with 10^5^ CFU/100 μL of wild-type parental BCG Pasteur or *ribH*-OE BCG strains. Control mice were sham immunized with saline. Six weeks after immunization with saline, BCG, or *ribH*-OE BCG, mice were challenged with M. tuberculosis (∼200 to 400 bacilli) by the aerosol route, and CFU were enumerated at 5 weeks postinfection along with gross pathological evaluation using the methods described above.

### (iii) Statistical analysis.

For comparisons between groups, one-way analysis of variance (ANOVA) with Tukey’s posttest was employed. Differences were considered significant when *P* was <0.05. For statistical analysis, Prism 5 software (GraphPad Software, Inc.) was used.
