# Supplementary material for: Augmentation of the Riboflavin-Biosynthetic Pathway Enhances Mucosa-Associated Invariant T (MAIT) Cell Activation and Diminishes Mycobacterium tuberculosis Virulence
Source: mBio. 2022 Feb 15;13(1):e03865-21. doi: 10.1128/mbio.03865-21 (PMC8844931; doi:10.1128/mbio.03865-21)
Supplement: TABLE S1 [file mbio.03865-21-st001.pdf]

**Supplementary Table 1: Gene description and List of primers used in the study**

| Primer Name | Gene number and Gene name | Gene description | Gene essentiality * | Sequence | Size of primer (bp) |
|---|---|---|---|---|---|
| NdeI_ribA2_F | *Rv1415* (MT1458) *ribA2* | bifunctional riboflavin biosynthesis GTP cyclohydrolase II/3,4-dihydroxy-2-butanone 4-phosphate synthase | Essential | GGGGCATATGATGACGAGGTTGGACTCCGT | 30 |
| MluI_ribA2_R | | | | GGGGACGCGTTCACAAGGCACCGCCGAATTCTC | 33 |
| NdeI_ribF_F | *Rv2786c* (MT2856) *ribF* | bifunctional riboflavin kinase/FMN adenylyltransferase | Essential | GGGGCATATGGTGCGCCGTAGGCTTGCGATCGT | 33 |
| MluI_ribF_R | | | | GGGGACGCGTTCAACCCGTAGACAACAGGTCAC | 33 |
| NdeI_ribG_F | *Rv1409* (MT1453) *ribG* | bifunctional riboflavin biosynthesis diaminohydroxyphosphoribosylaminopyrimidine deaminase/5-amino-6-(5-phosphoribosylamino) uracil reductase | Essential | GGGGCATATGATGAACGTGGAGCAGGTCAAGAG | 33 |
| PstI_ribG_R | | | | GGGGCTGCAGCTAACGAGCCACCAAGCTCAGCA | 33 |
| NdeI_ribH_F | *Rv1416* (MT1459) *ribH* | 6,7-dimethyl-8-ribityllumazine synthase | Essential | GGGGCATATGGTGAAGGGTGGCGCCGGGGTGCC | 33 |
| MluI_ribH_R | | | | GGGGACGCGTTCACGAGTGAGCGCGCAGCTCGC | 33 |
| Kan(F) | $Kan^r$ | Kanamycin resistance gene | | GAGAAAACTCACCGAGGCAG | 20 |
| Kan(R) | | | | GTATTTCGTCTCGCTCAGGC | 20 |

* Gene numbers; Rv- notation is used to reflect gene number in *M. tuberculosis H37Rv*; MT- notation is used to reflect gene number in *M. tuberculosis* CDC1551. However, at sequence level riboflavin biosynthetic genes (ribA2, ribF, ribG and ribH) of H37Rv bear 100% identity to respective genes in CDC1551. The riboflavin biosynthetic pathway is represented as per the information available in kegg pathway (https://www.genome.jp/kegg-bin/show_pathway?mtu00740 for *M. tuberculosis* H37Rv and https://www.genome.jp/pathway/mtc00740 for *M. tuberculosis* CDC1551)

* * Gene essentiality information is based on https://webhost.nts.jhu.edu/target/Default.aspx. Bosch, B *et al*, 2021; DeJusus, MA et al, 2017.
