# Supplementary material for: Augmentation of the Riboflavin-Biosynthetic Pathway Enhances Mucosa-Associated Invariant T (MAIT) Cell Activation and Diminishes Mycobacterium tuberculosis Virulence
Source: mBio. 2022 Feb 15;13(1):e03865-21. doi: 10.1128/mbio.03865-21 (PMC8844931; doi:10.1128/mbio.03865-21)
Supplement: FIG S1 [file mbio.03865-21-sf001.pdf]

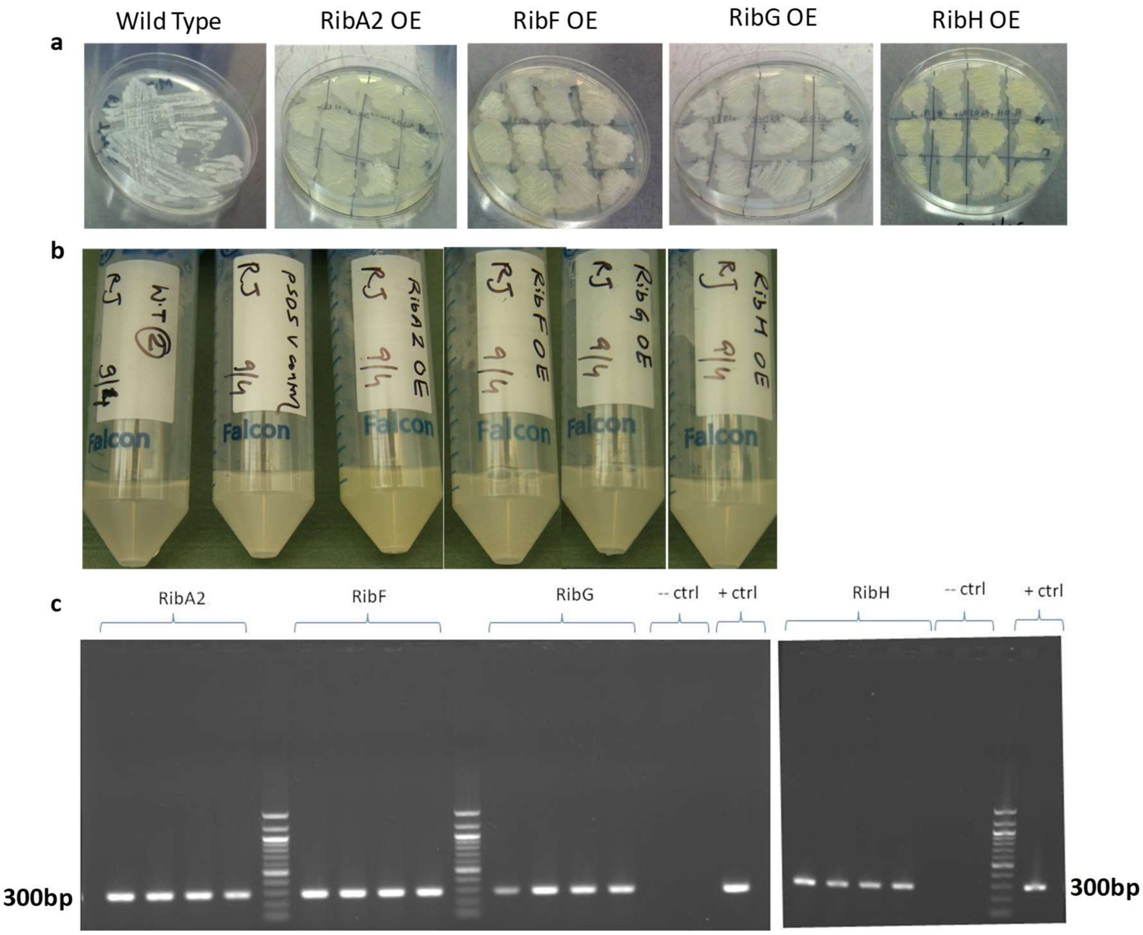

Supplementary Figure 1. Characterization of recombinant strains of *M. smegmatis.* (A) Morphological features of recombinant clones over-expressing *ribA2*, *ribF*, *ribG* and *ribH* genes of *M. tuberculosis* in *M. smegmatis.* Recombinant bacteria over-expressing (OE) *ribA2* and *ribH* appear to be relatively more pigmented (yellowish) than the wild type or recombinant strains over-expressing *ribF* or *ribG*. (B) Colonies for each strain were inoculated into 7h9 broth and growth characteristics were determined for recombinant strains with respect to wild type *M. smegmatis*. All the strains grew similarly but RibA2 OE broth cultures were relatively more pigmented (yellowish) compared to other strains suggesting greater secretion of the riboflavin or related metabolites of the pathway with characteristic yellow color. (C) Screening of recombinant clones was also performed by colony PCR on DNA isolated from recombinant clones (four each) of ribA2 OE, ribF OE, ribG OE, ribH OE using Kanamycin resistance gene (kan^r) specific primers as shown in supplementary table 1. Wild type *M. smegmatis* DNA and No template control were used as negative controls. DNA from bacteria containing vector control and purified vector control plasmid carrying kan^r were used as the positive control. Amplification in clones or positive control results in a PCR product of 300bp.
