# Supplementary material for: Augmentation of the Riboflavin-Biosynthetic Pathway Enhances Mucosa-Associated Invariant T (MAIT) Cell Activation and Diminishes Mycobacterium tuberculosis Virulence
Source: mBio. 2022 Feb 15;13(1):e03865-21. doi: 10.1128/mbio.03865-21 (PMC8844931; doi:10.1128/mbio.03865-21)
Supplement: FIG S2 [file mbio.03865-21-sf002.pdf]

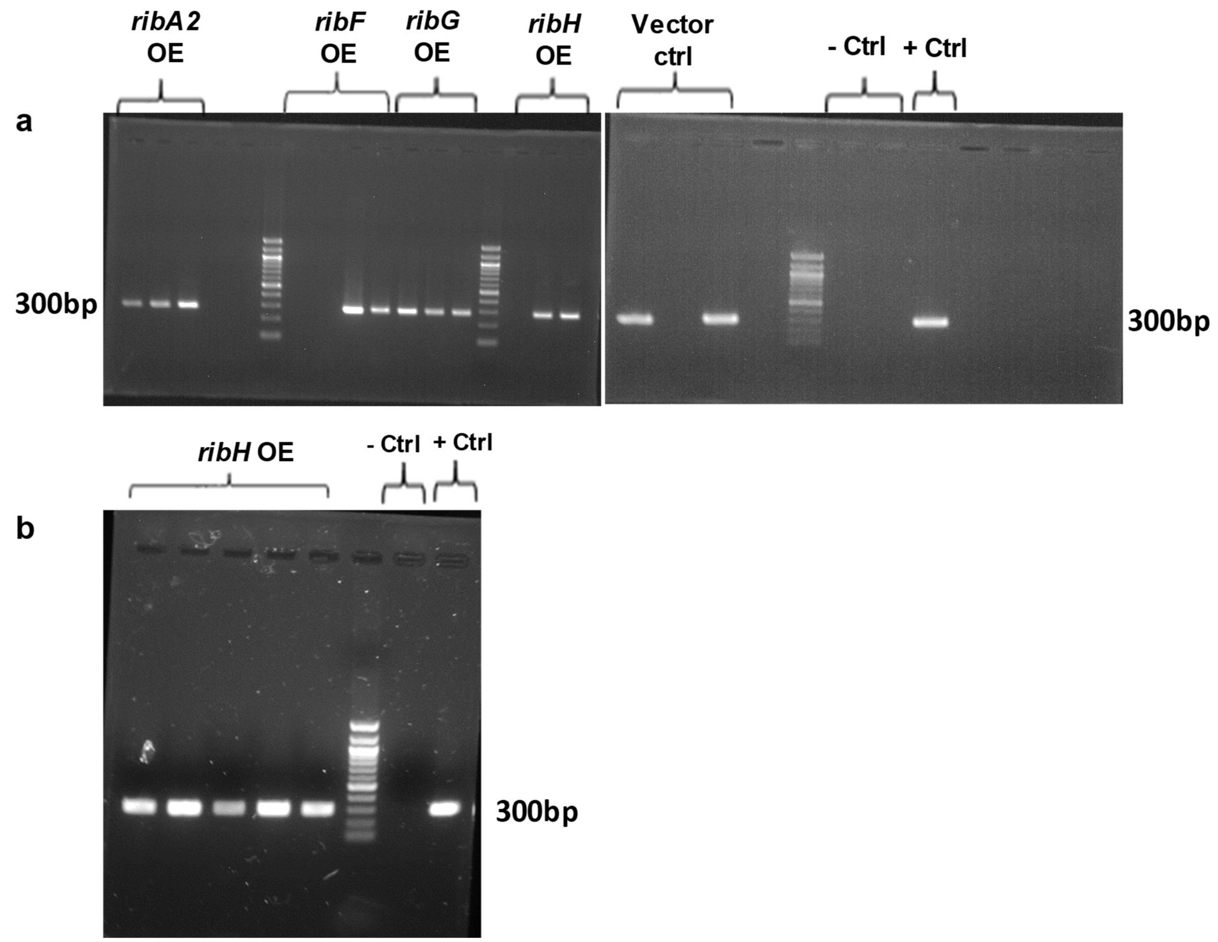

Supplementary Figure 2. Characterization of recombinant strains of (a) *M. tuberculosis and* (b) *M. bovis BCG.* (a) Screening of recombinant clones of *M. tuberculosis* over-expressing *ribA2* OE, *ribF* OE, *ribG* OE, *ribH* OE (B) Screening of recombinant clones of BCG over-expressing *ribH* OE. For screening colony PCR was performed on DNA isolated from recombinant clones using Kanamycin resistance gene (kan^r) specific primers. DNA isolated from Wild type *M. tuberculosis and* BCG and no template control were used as negative controls. DNA from bacteria containing vector control and/or purified vector control plasmid carrying kan^r were used as the positive control. Amplification in clones or positive control results in a PCR product of 300bp.
