# Supplementary material for: Augmentation of the Riboflavin-Biosynthetic Pathway Enhances Mucosa-Associated Invariant T (MAIT) Cell Activation and Diminishes Mycobacterium tuberculosis Virulence
Source: mBio. 2022 Feb 15;13(1):e03865-21. doi: 10.1128/mbio.03865-21 (PMC8844931; doi:10.1128/mbio.03865-21)
Supplement: FIG S3 [file mbio.03865-21-sf003.pdf]

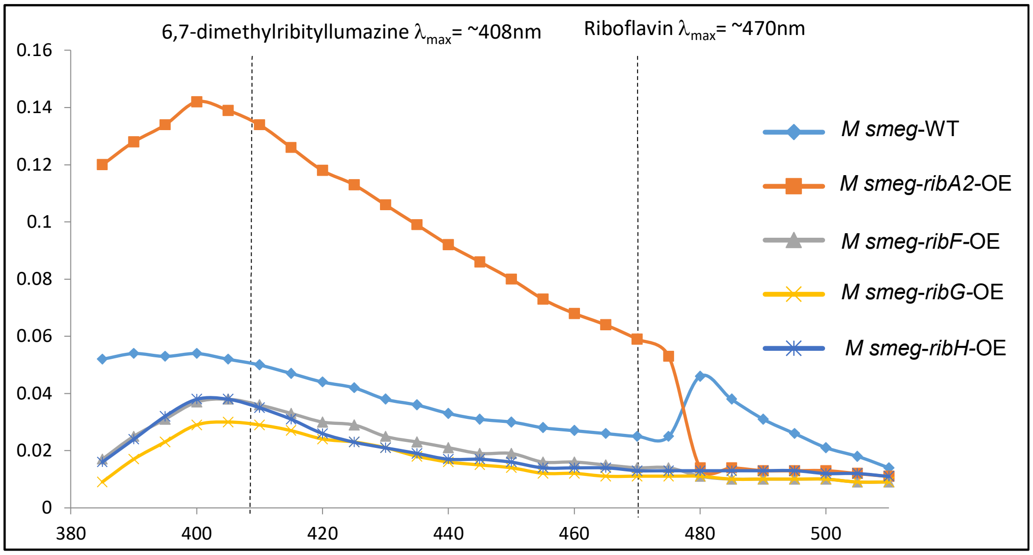

Supplementary Figure 3: Spectral analysis of culture supernatant of *M. smegmatis* strains over expressing genes of Riboflavin metabolites between 380nm-510nm. Compared to culture supernatant obtained from wild type *M. smegmatis*, culture supernatant obtained from *ribA2*-OE show a relatively higher absorbance between 380nm-475nm, with a peculiarly higher peak at wavelength corresponding to λ_{max} of 6,7-dimethylribityllumazine (λ_{max}= ~408nm) and riboflavin (λ_{max}= ~470nm) suggesting greater production and secretion of these metabolites on over-expression of first gene of the pathway i.e. *ribA2.* Interestingly, *ribH*-OE, despite showing pigmented colonies (yellowish), did not show any increase in secretion of the metabolites out into the broth compared to wild type *M. smegmatis*.
