# Supplementary material for: Augmentation of the Riboflavin-Biosynthetic Pathway Enhances Mucosa-Associated Invariant T (MAIT) Cell Activation and Diminishes Mycobacterium tuberculosis Virulence
Source: mBio. 2022 Feb 15;13(1):e03865-21. doi: 10.1128/mbio.03865-21 (PMC8844931; doi:10.1128/mbio.03865-21)
Supplement: FIG S4 [file mbio.03865-21-sf004.pdf]

a

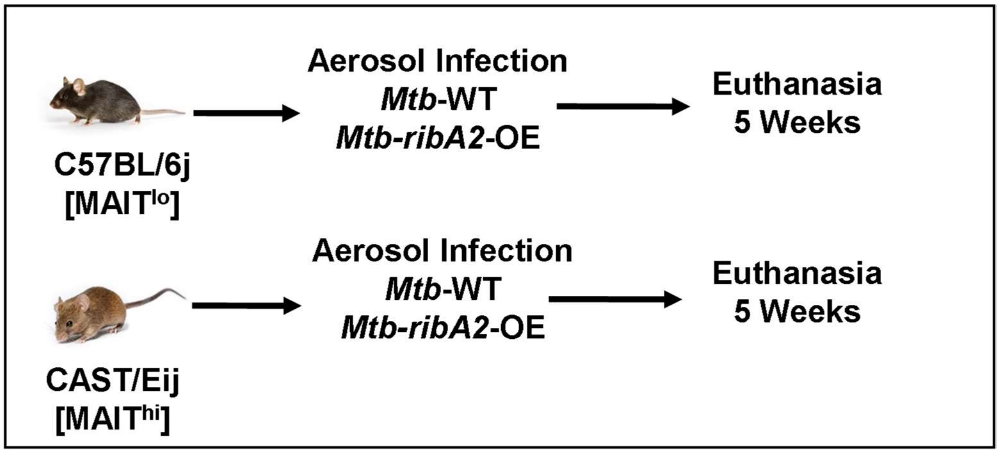

b

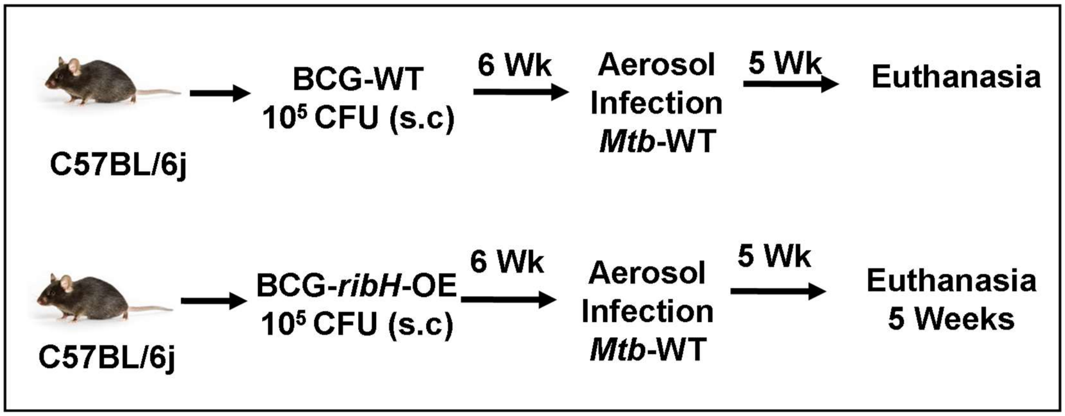

Supplementary Figure 4: Figure depicts (a) Study design of virulence study wherein virulence of Mtb-WT and Mtb-*ribA2*-OE was compared in $MAIT^{lo}$ (C57BL/6j) mice and in $MAIT^{hi}$ (CAST/Eij) mice at 5 weeks post-infection (b) Study design of vaccination studies carried out in C57BL/6j mice, wherein vaccine efficacy of BCG-ribH-OE was tested against aerosol infection with Mtb-WT.
