# Supplementary material for: Augmentation of the Riboflavin-Biosynthetic Pathway Enhances Mucosa-Associated Invariant T (MAIT) Cell Activation and Diminishes Mycobacterium tuberculosis Virulence
Source: mBio. 2022 Feb 15;13(1):e03865-21. doi: 10.1128/mbio.03865-21 (PMC8844931; doi:10.1128/mbio.03865-21)
Supplement: TABLE S2 [file mbio.03865-21-st002.pdf]

Supplementary Table 2: List of plasmids used in the study		
**Plasmid Name**	**Description**	**Source**
pSD5.hsp60	Mycobacterial expression plasmid with hsp60 plasmid	DasGupta, S.K, *et al*
pSD5.hsp60.Rv1415	Mycobacterial expression plasmid with hsp60 plasmid to over-express *Rv1415 (ribA2)*	This study
pSD5.hsp60.Rv2786c	Mycobacterial expression plasmid with hsp60 plasmid to over-express *Rv2786c (ribF)*	This study
pSD5.hsp60.Rv1409	Mycobacterial expression plasmid with hsp60 plasmid to over-express *Rv1409 (ribG)*	This study
pSD5.hsp60.Rv1416	Mycobacterial expression plasmid with hsp60 plasmid to over-express *Rv1416 (ribH)*	This study

Stover et al, first described development of extrachromosomal and integrative expression vectors with regulatory sequences for heat-shock proteins (hsp60) of BCG to allow expression of foreign antigens in BCG. Later on similar strategies was employed by DasGupta *et al* to develop a series of expression plasmids for making recombinant BCG and to study mycobacterial gene regulation. In this study, expression plasmids used was the one developed by DasGupta *et al*.
