# Supplementary material for: Augmentation of the Riboflavin-Biosynthetic Pathway Enhances Mucosa-Associated Invariant T (MAIT) Cell Activation and Diminishes Mycobacterium tuberculosis Virulence
Source: mBio. 2022 Feb 15;13(1):e03865-21. doi: 10.1128/mbio.03865-21 (PMC8844931; doi:10.1128/mbio.03865-21)
Supplement: TABLE S3 [file mbio.03865-21-st003.pdf]

Supplementary Table 3. List of bacterial Strains used in the study.	
**Name of the strain**	**Description**
***M. smegmatis* strains**	
Msmeg-WT	Wild type *M. smegmatis mc^{2}155**
Msmeg-ribA2-OE	*M. smegmatis mc^{2}155* strain over expressing *ribA2* (*Rv1415*)
Msmeg-ribF-OE	*M. smegmatis mc^{2}155* strain over expressing *ribF* (*Rv2786c*)
Msmeg-ribG-OE	*M. smegmatis mc^{2}155* strain over expressing *ribG* (*Rv1409*)
Msmeg-ribH-OE	*M. smegmatis mc^{2}155 strain* over expressing *ribH*(*Rv1416*)
**M. smegmatis* mc^{2}155 strain has been referred to as the wild type for the purpose of simplifying the nomenclature of the recombinant strains developed thereof in the study from this parent strain. The strain was originally reported by Panas *et al* and was shown to carry a mutation in *EptC* gene, the loss of which conferred the property of efficient plasmid transformation to *M. smegmatis* and hence forth provided a fast growing surrogate model organism to study fundamental cellular processes in mycobacteria.	
***M. tuberculosis* strains**	
Mtb-WT	Wild type *M. tuberculosis* CDC1551
Mtb-*ribA2*-OE	*M. tuberculosis* CDC1551 strain over expressing *ribA2* (*Rv1415*)
Mtb-*ribF*-OE	*M. tuberculosis* CDC1551 strain over expressing *ribF* (*Rv2786c*)
Mtb-*ribG*-OE	*M. tuberculosis* CDC1551 strain over expressing *ribG* (*Rv1409*)
Mtb-*ribH*-OE	*M. tuberculosis* CDC1551 strain over expressing *ribH*(*Rv1416*)
***M. bovis* BCG Pasteur strains**	
BCG-WT	*M. bovis* BCG Pasteur strains
BCG-*ribH*-OE 1 and 2	*M. bovis* BCG Pasteur strains over expressing *ribH* (*Rv1416*); 1 and 2 notation represents 2 clones of this strain that were tested in this study.

References in Supplementary Materials:

1. Stover, C., de la Cruz, V., Fuerst, T. *et al.* 1991. New use of BCG for recombinant vaccines. Nature **351:** 456–460.

2. DasGupta SK, Jain S, Kaushal D, Tyagi AK. 1998. Expression systems for study of mycobacterial gene regulation and development of recombinant BCG vaccines. Biochem Biophys Res Commun 246:797-804.

3. Panas MW, Jain P, Yang H, Mitra S, Biswas D, Wattam AR, Letvin NL, Jacobs WR. 2014. Noncanonical SMC protein in Mycobacterium smegmatis restricts maintenance of Mycobacterium fortuitum plasmids. Proceedings of the National Academy of Sciences 111:13264-13271
